# Decreased sleep is linked longitudinally and directionally to alterations in the brain’s intrinsic functional architecture

**DOI:** 10.1016/j.dcn.2025.101668

**Published:** 2025-12-31

**Authors:** M. Fiona Molloy, Aman Taxali, Mike Angstadt, Katherine Toda-Thorne, Katherine L. McCurry, Alexander Weigard, Omid Kardan, Camille Lehrmann, Joshua Vens, Cleanthis Michael, Mary M. Heitzeg, Chandra Sripada

**Affiliations:** a Department of Psychiatry, University of Michigan, Ann Arbor, MI, USA; b Department of Psychology, University of Michigan, Ann Arbor, MI, USA

**Keywords:** Sleep, Adolescent Brain Cognitive Development Study, Adolescence, Resting state, FMRI, Somatomotor connectivity, Longitudinal change

## Abstract

Previous cross-sectional studies demonstrated that reduced sleep is associated with widespread changes in the brain’s intrinsic functional architecture. The present study extends this work by clarifying links between sleep and the developing brain during adolescence both *longitudinally* (across two years) and *directionally* (does reduced sleep cause connectivity changes or are connectivity changes the cause of reduced sleep?). Our novel approach combines the Adolescent Brain Cognitive Development (ABCD) Study, a longitudinal observational study of 11,878 youth, and a second sample of 76 adult participants scanned after a typical night of sleep and after a sleep deprivation causal manipulation. First, in the ABCD dataset, we identified a robust and generalizable neurosignature of reduced sleep. Second, in an independent sample of ABCD participants, we demonstrate that greater reductions in sleep duration across two years are significantly related to greater expression of this neurosignature. Third, in the sleep deprivation dataset, we show that expression of the ABCD reduced sleep neurosignature is significantly increased within individuals following sleep deprivation, and that neurosignatures of reduced sleep from the two samples exhibit significant spatial correspondence. These results clarify links between sleep and the developing brain and provide novel evidence that changes in sleep produce characteristic brain functional connectivity changes across adolescence.

## 1 Introduction

Sleep is essential for mental and physical health (Medic et al., 2017), and inadequate sleep has been associated with reduced productivity, accidents, and reduced well being (Hillman et al., 2018). Adolescents may be particularly impacted by negative effects from inadequate sleep. Sleep plays a key role in healthy brain development (Galván, 2020, Lokhandwala and Spencer, 2022), and reduced duration and quality of sleep in adolescence is linked to worse academic and social outcomes as well as diverse psychiatric symptoms spanning both internalizing and externalizing domains (McCurry et al., 2024). Yet most adolescents receive inadequate sleep (Lewien et al., 2021) leading the National Sleep Foundation to deem adolescent sleep deprivation a public health epidemic (Buxton et al., 2015). There is thus substantial interest in identifying how reduced sleep affects the developing brain, especially during adolescence, which in turn can inform early risk identification and novel interventions.

The human brain is a complex network of interconnected regions, and coordinated activity across these regions underlies social, emotional, and cognitive processes and shapes risk for psychiatric illness (Sporns, 2011, van den Heuvel and Sporns, 2019). Task-free “resting-state” functional magnetic resonance imaging (fMRI) measures spontaneous neural activity and the strength of communication between brain regions can be described by computing coactivation (functional connectivity) (Biswal et al., 1995, Friston, 2011, Friston et al., 1993, Smith et al., 2013). Numerous resting-state functional connectivity studies have shown the brain is organized into multiple intrinsic networks that are linked to individual differences in cognition, emotion, and behavior (Castellanos et al., 2013, Woo et al., 2017).

Using resting-state functional connectivity, researchers have described a number of alterations associated with sleep duration, implicating diverse brain regions and functional networks. Most previous studies used experimental methods in which subjects were scanned before and after sleep deprivation, and these studies found alterations throughout the brain including the default mode network (Xu et al., 2018, Ben Simon et al., 2017, Yeo et al., 2015, De Havas et al., 2012, Li et al., 2023, Yu et al., 2025, Zhang et al., 2025, Ning et al., 2022, Chen et al., 2018), executive/control networks (Ben Simon et al., 2017, Yeo et al., 2015, De Havas et al., 2012, Ning et al., 2022, Tian et al., 2024, Liu et al., 2014), insula (Qi et al., 2021a, Qi et al., 2021b, Peng et al., 2024a), cingulate (Qi et al., 2021a, Qi et al., 2021b, Peng et al., 2024a, Zhang et al., 2021), postcentral gyrus/somatomotor network (Zhang et al., 2025, Wang et al., 2021, Peng et al., 2024b, Chen et al., 2023), visual network (Yu et al., 2025, Cai et al., 2021, Weng et al., 2025, Zhang et al., 2025), and subcortical areas (Li et al., 2023, Qi et al., 2021a, Peng et al., 2024a, Zhang et al., 2021, Wang et al., 2021, Cai et al., 2021, Feng et al., 2024, Tian et al., 2022, Liu et al., 2024, Qi and Hu, 2025, Shao et al., 2013). However, there was substantial variability across studies in regions exhibiting significant differences, and mixed results on whether sleep deprivation increases or decreases connectivity in these areas. We summarize these studies in Appendix A.

Some of the inconsistency in results from previous experimental studies may arise from small sample sizes, which increases variability in detected effects (Marek et al., 2022), and the use of region of interest-based methods, which limit analysis to a modest number of prespecified regions. These problems can potentially be addressed by utilizing large consortium studies, such as the Adolescent Brain Cognitive Development^SM^ (ABCD) (Garavan et al., 2018) Study and the Human Connectome Project (HCP) (Van Essen et al., 2013), combined with whole-brain multivariate methods that aggregate small effects distributed throughout the brain. Recently, our group applied multivariate predictive modeling to 3173 subjects in the ABCD Study®. We found a robust neurosignature of sleep, and using leave-one-site-out cross-validation, we established this signature generalizes to unseen subjects. We additionally established increased within-network connectivity of somatomotor network (SMN) as a key network motif associated with reduced sleep duration. These results align with a study that applied comparable multivariate methods to the HCP dataset (Mummaneni et al., 2023), and are generally consistent with previous studies in ABCD that examined connectivity changes associated with sleep duration with univariate tests across pairs of brain networks (Mummaneni et al., 2023, Brooks et al., 2022, Michael et al., 2025, Turan et al., 2025, Yang et al., 2022).

While the preceding findings from large consortium studies are intriguing, especially findings linking enhanced within-network somatomotor network (often considered a primary network) connectivity with reduced sleep duration, they leave additional key questions unanswered. One question concerns longitudinal change. Youths’ sleep patterns exhibit variability over time. Yet nearly all prior studies examined relations between sleep duration and brain connectivity at a single timepoint. It is unknown whether neurosignatures of brain connectivity that have a robust and generalizable link with reduced sleep duration *cross-sectionally* are additionally linked to changes in sleep duration *longitudinally*, especially across extended intervals of time spanning multiple years.

A critical related question concerns how to interpret functional connectivity neurosignatures of sleep duration directionally. That is, is the presence of the brain connectivity pattern identified in neurosignatures associated with reduced sleep a *consequence* of reduced sleep duration, or are these connectivity patterns a *contributor* to reduced sleep? Sleep is a critical node in a complex nomologic network linking social contexts, connectivity patterns of the maturing brain, and diverse social, academic, and psychopathological outcomes, making the preceding questions about longitudinal changes and directional effects particularly pressing for informing interventions that can promote healthy brain development. Thus, in the present study, we sought to take an important first step towards addressing these key questions.

Here, we used multimodal assessment of sleep coupled with multivariate predictive modeling to build a brain-wide neurosignature of reduced sleep at a single timepoint in ABCD. Next, to help address our first question about longitudinal change, we identified 1574 subjects in ABCD in the baseline and year-2 sample who did not contribute to training the neurosignature of sleep duration. We leveraged multilevel mixed effects modeling to precisely quantify the relationship between changes in sleep duration across the two timepoints and changes in expression of our neurosignature of reduced sleep across those two timepoints.

To help address the second question about directionality of effects, we utilized a novel method we dub “little data informs big data”. Neuroimaging studies that scan subjects before and after causal manipulation of sleep duration are highly resource intensive and challenging to undertake, and thus they are invariably relatively modest in size. On the other hand, consortium-size studies such as ABCD are ideal for training whole-brain multivariate predictive models of sleep, as these brain-wide methods benefit greatly from large sample size (Marek et al., 2022). To gain some advantages of both approaches while illuminating the question of directionality, we “transported” our multivariate neurosignature of reduced sleep duration learned in the ABCD sample to a separate sample of 76 adults in the Stockholm Sleepy Brain Study who underwent a sleep manipulation in which they were scanned after a night of typical sleep and after a night of sleep deprivation (3 h or less of sleep) (Nilsonne et al., 2017). Specifically, we calculated expression of the ABCD reduced sleep neurosignature in each subject in the Stockholm sample in each scan after a night of typical sleep and after sleep deprivation. While this method cannot definitively *prove* a causal relationship, it can *provide evidence* about the direction of the relationship between reduced sleep and brain connectivity patterns. We hypothesized that if the connectivity patterns in the ABCD neurosignature are directionally a *consequence* of reduced sleep, then expression of this neurosignature should be reduced in the Stockholm sample in the scan after experimentally-induced sleep deprivation compared to the scan without sleep deprivation.

## 2 Methods

### 2.1 Sample and data

The ABCD Study is a multisite longitudinal study with 11,878 children between 9 and 10 years of age from 21 sites across the United States. The study conforms to the rules and procedures of each site’s Institutional Review Board, and all participants provide informed consent (parents) or assent (children). The ABCD data repository grows and changes over time. The ABCD data used in this report came from ABCD Release 5.1 (http://dx.doi.org/10.15154/z563-zd24).

The Stockholm Sleepy Brain study involved a within-subject sleep deprivation manipulation and was used to assess directional relationships between reduced sleep and brain connectivity. This sample contained 86 adults scanned on 2 days with one day being after a night of normal sleep and the other after a night of sleep deprivation (instructed to sleep for 3 h). These data have been presented extensively (Nilsonne et al., 2017, Åkerstedt et al., 2020, Tamm et al., 2017, Tamm et al., 2019, Tamm et al., 2020).

### 2.2 Data acquisition and fMRI preprocessing

In the ABCD Study, high spatial (2.4 mm isotropic) and temporal resolution (TR = 800 ms) resting-state fMRI was acquired in four separate runs (5 min per run, 20 min total). Preprocessing was performed using fMRIPrep v1.5.0 (Esteban et al., 2019). Briefly, T1-weighted (T1w) and T2-weighted images were run through recon-all using FreeSurfer v6.0.1, spatially normalized, rigidly coregistered to the T1, motion corrected, normalized to standard space, and transformed to CIFTI space. Global signal regression was not performed in light of recent work indicating while physiological factors do contribute to the global signal, these influences are related to arousal, psychopathology, and cognition, not simply noise (Liu et al., 2017, Uddin, 2020, Yuan et al., 2025). See Supplement for details.

The Stockholm dataset consisted of high spatial resolution data (2.25 x 2.25 x 3 mm, 2.4 s TR). Two resting state runs of 8 min were collected during each scanning session. The data was processed similarly to the ABCD data using fMRIPrep v21.0.3 with outputs in CIFTI space.

### 2.3 Inclusion/exclusion

There are 11,878 participants in the ABCD Release 5.1 dataset, which contains neuroimaging data at baseline (ages 9–10) and year-2 follow-up (ages 11–12). Our ABCD multivariate predictive modeling analysis for generating a neurosignature of reduced sleep relied on year-2 data, which included three measures of sleep duration required for producing a sleep factor. Participants were excluded for: failing ABCD raw quality control (QC), insufficient number of resting-state runs each ≥ 4 min after censoring frames with FD > 0.5 mm, failing visual QC of registrations and normalizations, and missing data required for analysis (see Supplement for additional details). This left N = 2991 participants across 21 sites who had complete data on all sleep measures for examining how brain architecture relates to sleep.

For our ABCD longitudinal analysis, we examined expression of the neurosignature described in the previous paragraph in a separate group of subjects who had available imaging data and sleep measures in both the baseline and year-2 timepoints. This resulted in N = 1574 unique subjects for the longitudinal analysis. Demographic characteristics of included participants are shown in Supplementary Table S1.

For the Stockholm dataset, subjects were included if they had ≥ 4 min after censoring frames with FD> 0.5 mm in both runs. This left N = 76 participants for the Stockholm analysis (demographic characteristics of included participants are shown in Supplementary Table S2).

### 2.4 Connectome generation

For the ABCD sample, connectomes were generated for each functional run using the Gordon 333 parcel atlas (Gordon et al., 2016), augmented with parcels from high-resolution subcortical (Tian et al., 2020) and cerebellar (Diedrichsen et al., 2011) atlases. Volumes exceeding a framewise displacement threshold of 0.5 mm were marked to be censored. Covariates were regressed out of the time series in a single step, including: linear trend, 24 motion parameters (original translations/rotations + derivatives + quadratics), aCompCorr 5 CSF and 5 WM components and ICA-AROMA aggressive components, high-pass filtering at 0.008 Hz, and censored volumes. Next, correlation matrices were calculated for each run. Each matrix was then Fisher r-to-z transformed and then averaged across runs for each participant to yield their final connectome. For the Stockholm dataset, connectomes were generated in the same fashion from the fMRIPrep processed data.

### 2.5 Latent variable modeling for sleep duration

We constructed a latent variable for sleep duration by applying factor analysis to three measures:

*(Parent-report) Sleep Disturbance Scale for Children (SDSC)*:

The SDSC is a parent-reported measure consisting of 26 questions assessing six domains of sleep disturbances over the past six months using a 5-point Likert scale (Bruni et al., 1996). This scale has been used in other studies using the ABCD sample, is a valid and reliable measure of sleep disturbances, and fulfills psychometric requirements among childhood sleep scales. Here, we focused on the sleep duration item of this scale.

*(Child-report) ABCD Youth Munich Chronotype Questionnaire (MCTQ-C)*:

The MCTQ-C is a standardized self-reported measure consisting of 17 items that assess chronotypes, or diurnal preferences that manifest in personal sleep-wake rhythms, including sleep and wake schedules on school and school-free days (Zavada et al., 2005). This scale has been used in other studies using the ABCD sample. Here, we focused on the weighted average of sleep duration for school days and non-school days.

*(Objective measure) Fitbit Measure of Sleep Duration:*

Fitbit devices measure biobehavioral features, such as sleep and physical activity, continuously and unobtrusively. Youth wore a Charge 2 Fitbit over 21 days and were instructed to remove it only when charging and bathing (Bagot et al., 2018). The Fitbit app was downloaded on either the youth or parent’s phone and participants were instructed to sync the Fitbit daily. *Fitabase* was used by each site to monitor and track Fitbit data. Sleep intervals were captured by an intrinsic device algorithm. Fitbit-based measures of sleep duration have been examined in other studies using the ABCD sample and have been shown to have adequate sensitivity in validation studies (Godino et al., 2020). Similar to the other two measures, we focused on the weighted average of sleep duration for weekdays and weekends.

### 2.6 Latent variable construction

**Fig. 1:**
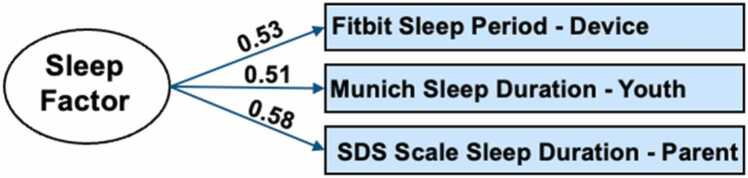
Factor Model of Sleep Duration. Path estimates reflect standardized factor loadings.

To construct a latent variable for sleep duration that triangulates across objective (Fitbit) and subjective (parent- and youth-report) assessments (Fig. 1, see Supplement for additional details), we conducted a factor analysis using the “fa” function from the psych package (version 2.3.3) in R (version 4.1.1). Since the model only has three indicators, it is just-identified; therefore, model fit indices are not available. The level of agreement/disagreement between our measures of sleep duration, is shown in Table 1, which displays zero-order Pearson correlations between each measure of sleep duration. Note that the results were also replicated in a sensitivity analysis using the weighted average of the sleep duration variables, as opposed to the sleep factor (Supplement §7).

**Table 1:** Zero-Order Correlations among Sleep Duration Variables. MCT-Q refers to the youth-reported Munich Chronotype Questionnaire, and SDSC refers to the parent-reported Sleep Disturbance Scale for Children.

**Sleep Variable**	Fitbit (weekdays)	Fitbit (weekends)	MCT-Q (weekdays)	MCT-Q (weekends)
Fitbit (weekdays)	-	-	-	-
Fitbit (weekends)	.43	-	-	-
MCT-Q (weekdays)	.32	.20	-	-
MCT-Q (weekends)	.19	.19	.30	-
SDSC (overall)	.30	.19	.31	.11

### 2.7 Multivariate predictive modeling

To quantify the multivariate relationship between connectomes and sleep duration, we used principal component regression (PCR) predictive modeling (Sripada et al., 2019, Sripada et al., 2020) (see Supplement and Supplementary Figure S1 for more details). Briefly, this method performs dimensionality reduction on the set of predictive features (i.e., edges in the connectome), fits a regression model on the resulting components (where the number of components is determined in nested cross-validation by selecting the smallest number of components that is not more than 1 standard error different from the minimum), and applies this model out-of-sample using leave-one-site-out cross-validation (LOSO-CV). We can then generate a neurosignature by fitting a model on the whole sample using the average selected number of components across folds and then multiplying the principal components with their beta weights to yield a single map that can generate predicted values. Predictive accuracy is reported as the cross-validated r-value (r_cv_), averaging across held-out sites. For visualization, we apply the Haufe transform to the beta weights (Haufe et al., 2014), which has been shown to better capture spatial localization of effects. We control for multiple covariates including sex assigned at birth, age, age-squared, mean FD, and mean FD-squared. In sensitivity analyses (reported in Supplement §6), we also controlled for parent-reported race/ethnicity, a social construct, as a proxy to account for differences in exposure to personal and systemic racism, disadvantage, and opportunity among people of color in the US, because these variables were not directly measured (Pager and Shepherd, 2008). Statistical significance was determined with non-parametric permutation tests, using the procedure of Freedman and Lane (Freedman and Lane, 1983) to account for covariates. Exchangeability blocks were used to account for twin, family, and site structure and were entered into Permutation Analysis of Linear Models (Winkler et al., 2014) to produce permutation orderings.

### 2.8 Longitudinal analysis

We examined expression of the ABCD neurosignature of reduced sleep in a disjoint ABCD sample with no overlapping subjects who had imaging data from baseline and year-2 timepoints.

To obtain expression scores, we projected the ABCD reduced sleep neurosignature onto the connectomes for each subject in this Longitudinal Sample via a vector dot product. More specifically, each connectome from each subject at baseline and year-2 was separately projected onto the ABCD reduced sleep signature by a vector dot product operation, which yields an expression score for each connectome reflecting the degree to which it expresses that neurosignature. We next examined whether differences in subjects’ sleep duration between baseline and year-2 predicted differences in their expression scores for the reduced sleep neurosignature across these same timepoints. More specifically, we entered change in expression of the neurosignature as an outcome variable in a linear mixed effects model with the change in sleep duration as a fixed effect predictor and ABCD site as a random effect and controlling for: age difference (linear and quadratic), mean motion at each timepoint (linear and quadratic), and sex at birth.

### 2.9 Quantifying expression of the ABCD reduced sleep neurosignature in the Stockholm Sleepy Brain Study sample

We next examined the expression of the ABCD neurosignature of reduced sleep (calculated using the methods described in §6 above) in subjects in the Stockholm Sleepy Brain Study sample both after a typical night of sleep and after a sleep deprivation manipulation. To obtain expression scores, we projected the ABCD reduced sleep neurosignature onto the connectomes for each subject of the Stockholm Sleepy Brain Study via a vector dot product. Expression scores were calculated separately for the post-typical sleep and post-sleep deprivation connectomes, then the change in these expression scores was used to assess the directional effect of sleep deprivation. We calculated differences in expression of this neurosignature post-typical sleep and post-sleep deprivation for each individual and assessed significance with a linear model controlling for sex, age group (young/old), and motion on each scan.

### 2.10 Quantifying spatial correspondence of the ABCD reduced sleep neurosignature and Stockholm Sleepy Brain Study sleep deprivation neurosignature

We generated a neurosignature of sleep deprivation from the 76-subject Stockholm Sleepy Brain Study using methods that parallel construction of the reduced sleep neurosignature in ABCD. We first regressed sex, age group (young/old), and motion at each scan out of the connectomes, then performed a principal components analysis of the residualized data, and then calculated differences in principal component scores. We averaged the differences across subjects, then multiplied them with the first 21 principal components (the same number used in the ABCD dataset) to get a single spatial map. To quantitatively assess the spatial correspondence of the ABCD reduced sleep neurosignature and Stockholm Sleepy Brain Study sleep deprivation neurosignature, we calculated the spatial correlation across connections between these neurosignatures. To assess statistical significance of this observed correlation, we used non-parametric permutation tests. We randomly shuffled sleep factor scores across subjects in the ABCD study 10,000 times and calculated neurosignatures of permuted sleep factor scores at each iteration. We in addition, we randomly shuffled “typical sleep” and “sleep deprivation” labels in the Stockholm data, and calculated neurosignatures at each iteration. These permuted neurosignatures, by construction, lack any relationship to sleep duration or sleep deprivation, respectively. At each iteration of the 10,000 permutations, we calculated the spatial correlation between a permuted ABCD neurosignature and permuted Stockholm neurosignature, yielding a distribution of spatial correlations under the null hypothesis that there is no relationship between the neurosignatures.

## 3 Results

### 3.1 Multimodal sleep assessment and multivariate predictive modeling identify a brain-wide functional connectivity neurosignature robustly related to shorter sleep duration

**Fig. 2:**
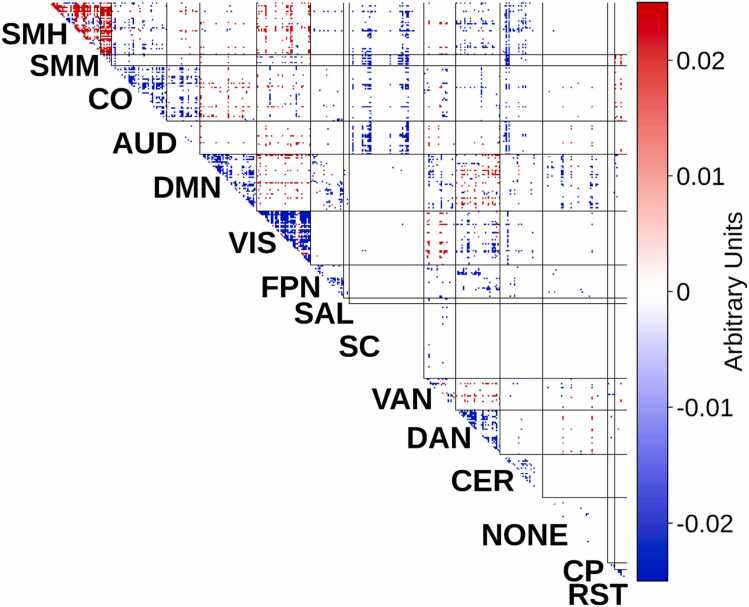
Neural Signature of Shorter Sleep Duration in the ABCD Study. The figure depicts an importance map, which captures brain-wide functional connections that are linked to shorter sleep duration, weighted by their importance. The sign indicates whether each connection is positively or negatively related to shorter sleep duration. This map shows a prominent role for connections in the somatomotor network, which showed stronger within-network connectivity with shorter sleep duration. There was in addition reduced within-network connectivity in the visual network. Network label abbreviations: SMH=somatomotor medial (hand), SMM=somatomotor lateral (mouth), CO=cingulo-opercular, AUD=auditory, DMN=default mode network, VIS=visual, FPN=frontoparietal network, SAL=salience, SC=subcortical, VAN=ventral attention network, DAN=dorsal attention network, CER=cerebellar, NONE=no assignment, CP=cinguloparietal, RST=retrosplenial temporal.

We constructed a multimodal measure of sleep duration combining parent-rated, child-rated, and Fitbit metrics in 2991 children drawn from the ABCD year-2 data. We next used brain-wide functional connectivity patterns as predictors in a multivariate predictive modeling framework to predict shorter sleep duration, to quantify predictivity and ensure generalizability to new participants. The out-of-sample multivariate link between shorter sleep duration and brain-wide functional connectivity was r_cv_ = 0.31 (averaging across sites), p_PERM_ < 0.0001. Visual inspection of this “reduced sleep” predictive neurosignature (Fig. 2) identified prominent elevation in intra-network connectivity in the somatomotor network and reduction of intra-network connectivity in the visual network with shorter sleep duration.

### 3.2 Longitudinal analysis across the ABCD baseline and year-2 sample demonstrates changes in sleep duration are linked to changes in expression in the “reduced sleep” neurosignature

Having identified a robust neurosignature of reduced sleep duration in a subsample of year-2 ABCD subjects, we next examined the expression of this neurosignature across the ABCD baseline and year-2 samples. Importantly, for this analysis, we leveraged a disjoint set of 1574 ABCD subjects who did not contribute to training the neurosignature described in the previous section and who had parent-rated sleep duration across both timepoints (Fitbit and child-rated could not be used as they were not available in ABCD baseline data).

After controlling for covariates, we found that in this ABCD longitudinal sample, changes in sleep duration were highly significantly associated with changes in expression of the reduced sleep duration neurosignature (standardized beta=0.10, *p* = 0.000050, Table 2). Specifically, decreased sleep was associated with increased expression of the neurosignature of reduced sleep.

**Table 2:** Longitudinal ABCD baseline and year-2 sample model summary.

**Effect**	Standardized Beta	p-value
Neurosignature expression difference	0.1017	0.00005
Linear age difference	-0.0255	0.3083
Quadratic age difference	0.0185	0.4581
Linear framewise displacement at baseline	-0.0172	0.5451
Quadratic framewise displacement at baseline	0.0425	0.096

### 3.3 Little-data-informs-big-data analysis: In the Stockholm Sleepy Brain Study sample of 76 subjects scanned after a typical night of sleep and after sleep deprivation, the “reduced sleep” neurosignature from ABCD is highly significantly more expressed after sleep deprivation

**Fig. 3:**
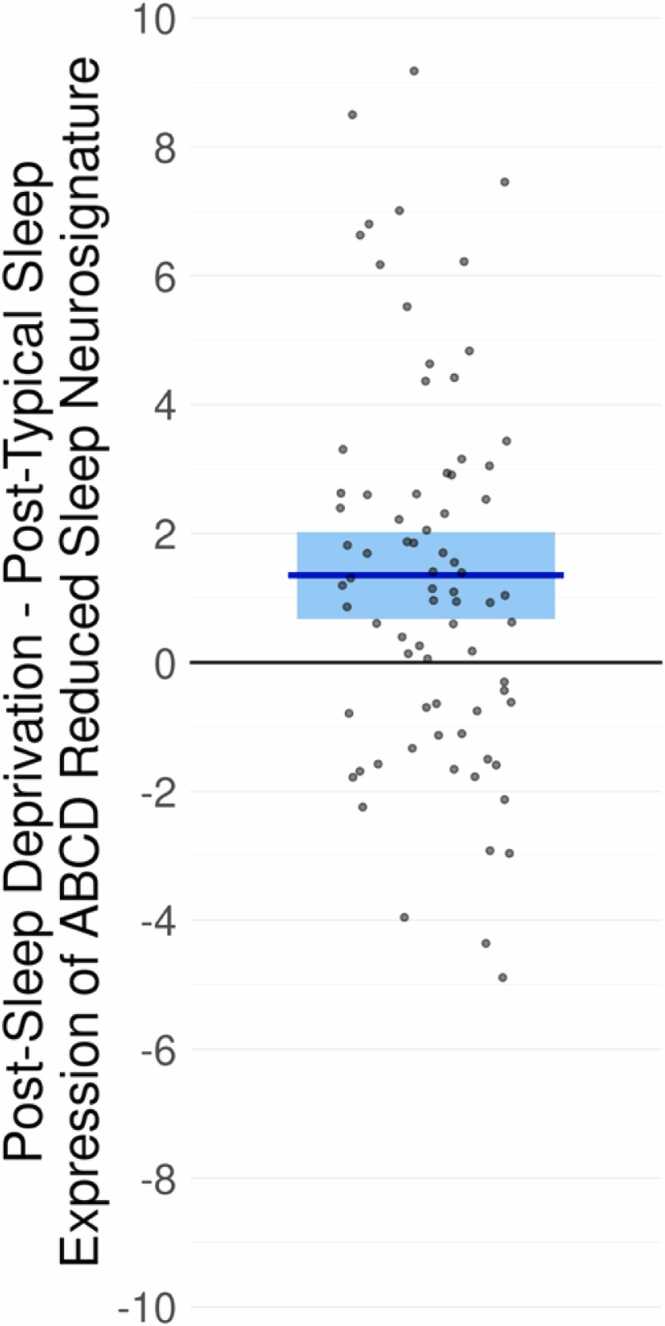
Increased expression of ABCD reduced sleep signature following sleep deprivation. Each point indicates the difference in the expression of the ABCD reduced sleep signature following sleep deprivation compared to the expression following a typical night of sleep for each individual in the Stockholm Sleepy Brain Study. Positive values indicate an increased neurosignature expression following sleep deprivation.

We next examined the directional effects of sleep deprivation on the expression of the reduced sleep neurosignature. For this analysis, we leveraged a separate sample of 76 adults who were scanned after a typical night of sleep and after a single night of sleep deprivation. For each scan for each subject, we calculated the expression of the ABCD reduced sleep neurosignature. We found expression of this neurosignature was significantly increased after sleep deprivation (mean=3.39, SE=0.340) compared to after a typical night of sleep (mean=2.036, SE=0.333), and the difference was highly statistically significant when controlling for sex, age group, and motion on each scan (T = 3.84, p = 0.00027, Fig. 3).

### 3.4 A comparison of the neurosignature of reduced sleep from ABCD and neurosignature of experimentally-induced sleep deprivation from the Stockholm Sleepy Brain Study reveals highly statistically significant spatial correspondence

**Fig. 4:**
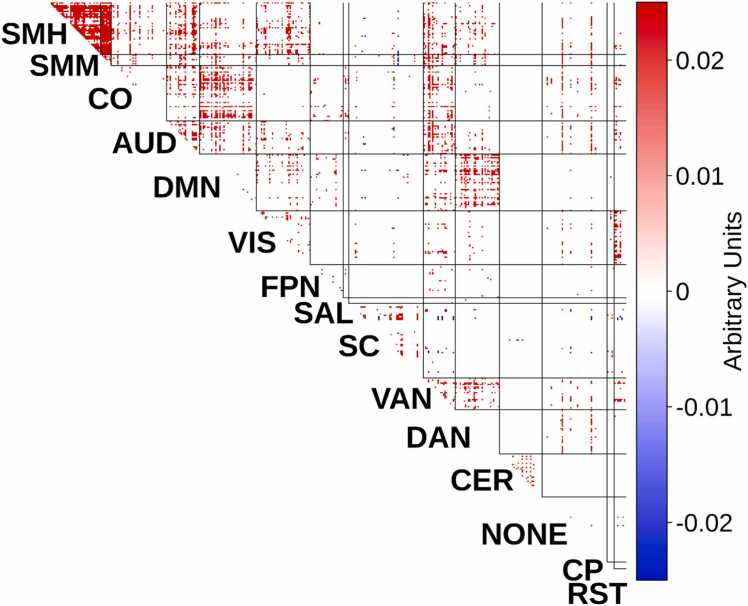
Neural Signature of Experimentally-Induced Sleep Deprivation in the Stockholm Study. The figure shows an importance map, which captures brain-wide functional connections that are linked to shorter sleep duration, weighted by their importance. The sign indicates whether each connection is positively or negatively related to shorter sleep duration. Network label abbreviations: SMH=somatomotor medial (hand), SMM=somatomotor lateral (mouth), CO=cingulo-opercular, AUD=auditory, DMN=default mode network, VIS=visual, FPN=frontoparietal network, SAL=salience, SC=subcortical, VAN=ventral attention network, DAN=dorsal attention network, CER=cerebellar, NONE=no assignment, CP=cinguloparietal, RST=retrosplenial temporal.

We next generated a neurosignature of sleep deprivation from the 76-subject Stockholm Sleepy Brain Study using methods that parallel construction of the reduced sleep neurosignature in ABCD. This neurosignature is shown in Fig. 4. In qualitatively comparing the Stockholm sleep deprivation neurosignature with the ABCD reduced sleep neurosignature (see Fig. 2), both neurosignatures show a prominent pattern of increased connectivity within the somatomotor network.

**Fig. 5:**
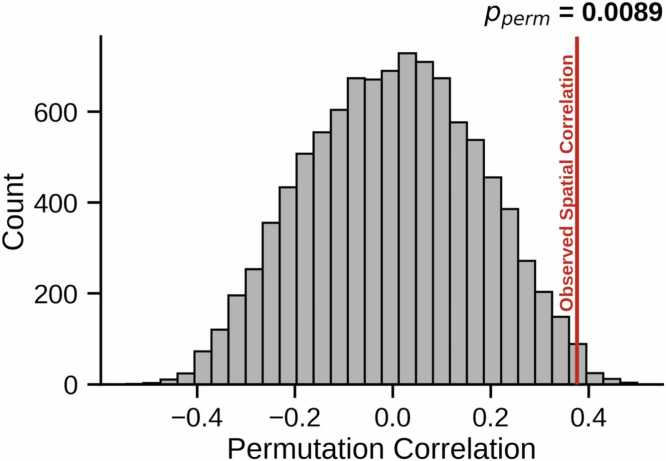
Spatial Correspondence Permutation Testing. We calculated the correlation across connections between the ABCD reduced sleep neurosignature and Stockholm Sleepy Brain Study sleep deprivation neurosignature. The observed spatial correspondence (r = 0.38) is denoted by the vertical red line, and the histogram shows the null distribution from 10,000 permutations. The two neurosignatures exhibit highly significant spatial correspondence (p_PERM_=0.0089).

To quantitatively assess the correspondence of these neurosignatures, we calculated the spatial correlation across connections between the ABCD and Stockholm neurosignatures, finding a correlation of 0.38. To assess statistical significance, we used non-parametric permutation tests, generating 10,000 permuted neurosignatures that lacked any relationship to sleep duration or deprivation, and calculating the spatial correlation between the ABCD and Stockholm permuted neurosignatures. This yields a distribution of spatial correlations under the null hypothesis that there is no relationship between the neurosignatures (Fig. 5). We found that the observed spatial correlation between the ABCD reduced sleep neurosignature and Stockholm sleep deprivation neurosignature exceeded all but 21 correlation scores in the permutation-based null distribution (*p*_PERM_=0.0089), suggesting that the observed level of spatial correspondence between the two neurosignatures is unlikely to have arisen by chance. These results were replicated in a sensitivity analysis that predicted a weighted average of sleep duration instead of the sleep factor (Supplement §7) and in another analysis that included race/ethnicity as a covariate (Supplement §6).

## 4 Discussion

Sleep is critical for social, academic, and occupational outcomes (Medic et al., 2017, McCurry et al., 2024), yet most adolescents receive less than the recommended amount of sleep (Lewien et al., 2021), and thus there is a need to understand linkages between sleep duration and the brain. There is substantial evidence that sleep duration has complex interlinkages with brain connectivity, but the directionality of this relationship and associated longitudinal changes remain unclear. This study extends previous work that linked sleep in adolescence and adulthood with brain-wide changes in connectivity (Mummaneni et al., 2023, Brooks et al., 2022, Michael et al., 2025, Turan et al., 2025, Yang et al., 2022), by combining the strengths of smaller, but highly controlled experimental studies and large-scale, longitudinal observational studies.

This study establishes four main results. First, using multimodal measurement of sleep and whole brain predictive modeling, we constructed a robust and generalizable neuromarker of sleep. Second, we demonstrate that this neurosignature is longitudinally related to sleep duration, such that changes in sleep duration are highly statistically significantly related to changes in neurosignature expression across a mean of 2.08 years. Third, using a novel “little data informs big data” approach, we show in the 76-subject Stockholm Sleepy Brain Study that expression of the ABCD neurosignature of reduced sleep is highly statistically significantly reduced in scans after a sleep deprivation manipulation compared to scans after a typical night of sleep. Fourth, we showed that neurosignatures of reduced sleep from the ABCD youth sample and the Stockholm adult sample exhibit highly statistically significant spatial correspondence. These results add to our understanding of how individual differences in sleep duration calibrate brain connectivity patterns longitudinally and directionally during adolescence, a critical window for brain maturation and social, emotional, and cognitive development.

We identified a robust and generalizable neurosignature of sleep using data-driven, multivariate predictive modeling. Leave-one-site-out cross-validation was used to test model fits with unseen subjects, demonstrating the generalizability of this marker. Additionally, in contrast to univariate approaches, multivariate methods have been found to yield more robust relationships to behavior in other domains (Spisak et al., 2023). This neuromarker revealed a prominent role for the SMN and visual network. This diverges from the predominant focus in previous sleep literature on higher-order networks, such as the frontoparietal and default mode networks. In contrast, the central role of primary sensory and motor networks demonstrates a major advantage of data-driven approaches, which can reveal involvement of understudied networks not typically associated with complex cognition. Importantly, our findings align with recent studies linking primary network connectivity to arousal (Laumann et al., 2015, Tagliazucchi and Laufs, 2014), and SMN connectivity to psychopathology (Kebets et al., 2019), sleep (Michael et al., 2025), and socioeconomic resources (Michael et al., 2024).

Using multiple time points in ABCD, we extended previously cross-sectional findings to draw a tighter link between this neurosignature and sleep duration in adolescence. We found a highly statistically significant relationship between changes in sleep duration across the two time points and changes in expression of the reduced sleep neurosignature across these timepoints. Critically, this relationship was found in a sample independent of that used to generate the neurosignature, further supporting the generalizability of these results. In this within-subject analysis, each subject acts as their own control, so this brain-sleep association is less likely to be affected by confounds, such as baseline differences in demographics or environmental exposures among the subjects. Our results thus provide among the strongest lines of evidence to date that changes in sleep over the course of years are associated with changes in brain connectivity.

We next investigated the *directional* effect of the ABCD reduced sleep neurosignature in relation to sleep duration leveraging the Stockholm Sleepy Brain Study. We found that the reduced sleep neurosignature was significantly more expressed within an individual following a causal sleep deprivation manipulation compared to a night of typical sleep. This result provides evidence that the connectivity patterns captured in the neurosignature are a *consequence* of reduced sleep. If the neurosignature was instead exclusively a cause of reduced sleep, that is, the identified connectivity patterns cause reduced sleep drive or make the person more activated and awake, then one would not expect expression of the neurosignature to be highly significantly increased after a sleep deprivation manipulation.

To be clear, while the preceding findings are suggestive, it must be emphasized that they are ultimately circumstantial and do not definitively settle the issue of causality. Two cautionary points are in order. First, the only way to definitively and comprehensively establish the causal directionality of the ABCD reduced sleep neurosignature involves experimentally manipulating levels of the neurosignature and examining subsequent effects on sleep duration. Of course, directly manipulating brain connectivity patterns in this way is neither scientifically possible or ethically acceptable, and thus our results fall short of what is required to definitively establish causal directionality. But most theorists agree that even in the absence of definitive experimental manipulations, there are a variety of ways to *provide evidence* for causal directionality, for example regression discontinuity methods, difference-in-difference methods, and Mendelian randomization, to name just a few. All of these methods add evidential weight in favor of causal hypotheses, without definitively establishing them. Like these other methods, the methods used in this study, in which we show that the reduced sleep neurosignature from ABCD is more expressed after sleep deprivation and is spatially correspondent to a sleep deprivation neurosignature, adds evidential weight in favor of a causal hypothesis that reduced sleep *produces* brain connectivity changes, but our results do not definitively establish this hypothesis.

Second, the ABCD neurosignature of reduced sleep could have bidirectional relations with sleep duration; it may both be a consequence of reduced sleep as well as a cause of reduced sleep. Nothing in our results directly supports this hypothesis, but critically nothing in our results necessarily rules out this possibility. Thus, while our results provide some (non-definitive) evidence that the connectivity patterns in the neurosignature are a consequence of reduced sleep, we cannot rule out the possibility that the neurosignature is *in addition* a cause of reduced sleep.

Finally, we found strong spatial correspondence between the Stockholm sleep deprivation neurosignature and ABCD reduced sleep neurosignature. This observation also supports the view that the ABCD reduced sleep neurosignature arises as a consequence of reduced sleep. The reason is that the Stockholm study involves experimenter manipulation (unlike ABCD which is an observational study), so patterns of brain connectivity changes from that study can be interpreted as being a consequence of reduced sleep. The fact that this Stockholm neurosignature is highly significantly spatially similar to the ABCD neurosignature thus provides evidence that the latter too likely reflects a consequence of reduced sleep. The same two caveats raised in the previous paragraph apply: 1. Evidence arising from spatial correspondence is suggestive and not definitive; 2. Our spatial correspondence result does not rule out the possibility that the ABCD reduced sleep neurosignature *also* operates in the other direction as a cause of reduced sleep duration.

The directional relationship our study supports, in which brain connectivity changes occur as a consequence of sleep deprivation, aligns with current theories of underlying mechanisms driving sleep-related changes in cognition and brain function. Recent studies using animal models of sleep deprivation have identified neuronal changes following experimentally-induced reduction in sleep confirming a causal effect of sleep deprivation on the brain. Importantly, these studies show that these brain changes are largely reversible following restoration of sleep (Zamore and Veasey, 2022). A similar pattern is observed in humans, where changes in resting state functional connectivity following acute sleep deprivation are restored after multiple nights of sufficient sleep (Chai et al., 2020). Recent work has indicated that this restoration of connectivity can also be modulated using stimulants, where functional connectivity profiles following methylphenidate use closely resemble those associated with sufficient sleep (Kay et al., 2025). Interestingly, the largest effects of stimulants were found in somatomotor networks, mirroring the prominence of SMN connectivity in the sleep neurosignatures found in the present study. These brain changes following sleep deprivation and reversal of these changes following sleep restoration or pharmacological intervention, are more consistent with these brain changes being a consequence of reduced sleep (rather than a precursor that causes reduced sleep), in alignment with the findings of the present study.

We observed a prominent association between reduced sleep/sleep deprivation and increased within-network SMN connectivity in both the ABCD and Stockholm Sleepy Brain datasets. This result contrasts with a previous focus in the sleep field on higher-order networks, especially default mode and frontoparietal networks. There is, however, growing recognition that the SMN supports a broader array of cognitive functions than exclusively motor control. According to recent models, the SMN becomes more integrated with other systems across development to coordinate neural computations associated with action planning and translate them into goal-directed behavior (Gu et al., 2015, Pines et al., 2022). For example, precision functional mapping indicates that regions of motor cortex are tightly interconnected with regions from the cingulo-opercular network, as part of a “somato-cognitive action network”, which integrates cognitive control processes with action (Gordon et al., 2023). Additionally, altered somatomotor connectivity has been associated with externalizing problems in meta-analyses (Dugré et al., 2022) and general psychopathology (the “p factor”) in a transdiagnostic adult sample (Kebets et al., 2019). These findings emphasize that complex cognition is an emergent property of interactions between multiple large-scale systems, with SMN regions playing a key mediating role, especially in domains including cognitive control and goal-directed action, both of which are disrupted by reduced sleep (Medic et al., 2017, Brooks et al., 2022).

We also observed an association between sleep duration and connectivity in the visual network in the ABCD dataset. This finding is consistent with previous studies conducted using different methodologies in ABCD (Mummaneni et al., 2023, Brooks et al., 2022) and HCP (Mummaneni et al., 2023). Experimental studies have also shown that reduced sleep can yield disruption in visual regions (Yu et al., 2025, Liu et al., 2014, Qi et al., 2021b, Cai et al., 2021, Weng et al., 2025, Zhang et al., 2025, Qi and Hu, 2025). In contrast, we did not find pronounced changes in the visual network following sleep deprivation in the Stockholm Study. Multiple factors might have contributed to this difference, including differences in the sample size (and thus power to detect effects), differences in the type of sleep reduction (chronic reduced sleep versus acute sleep deprivation), and differences in subject characteristics (adolescents versus adults). Of note, age-related differences have been reported in the effects of sleep deprivation on attention, wherein adolescents tend to exhibit larger deficits in visual attention task performance following reduced sleep than young adults (Lo et al., 2016). Our results point to connectivity changes in the visual network as a possible mechanistic link–a hypothesis that warrants specific testing in future work. However, while our observed connectivity results are suggestive of potential mechanisms underlying prior behavioral studies, we do not investigate any measures of attention in the present study, and cannot definitively confirm the differences in visual network results are solely attributable to age differences.

This study has several limitations and opens up potential directions for future research. First, it is important to reemphasize that our results concerning causal directionality of sleep-brain associations are suggestive and not definitive, as only direct causal manipulations of key variables can establish causal directionality. Second, this study relied on retrospective measures of sleep available in the ABCD study that were mostly collected annually. However, sleep quality and duration can fluctuate over much shorter intervals, including night-to-night, week-to-week, and weekday-to-weekends, which cannot be readily captured in the present study. Future studies should use more granular methods for tracking sleep-brain associations, such as dense within-subject imaging at relatively brief intervals for an extended duration. Third, we focused in this study on ABCD baseline and year-2 releases, corresponding to the ages of 9–10 and 11–12. Additional data releases are planned from ABCD over the next several years that will capture middle to late adolescence, and similar analyses performed on these later waves will paint a broader picture of the relation between sleep and brain connectivity over the course of adolescence.

In sum, this study leveraged the large, longitudinal, observational ABCD imaging sample and a separate smaller imaging sample with a sleep deprivation causal manipulation to clarify the longitudinal and directional effects of reduced sleep on brain connectivity patterns in youth. Our results deepen our understanding of how sleep calibrates the developing connectome and shapes behavioral, emotional, and psychopathological outcomes.

## CRediT authorship contribution statement

**Camille Lehrmann:** Writing – review & editing. **Joshua Vens:** Writing – review & editing. **Cleanthis Michael:** Writing – review & editing. **Heitzeg Mary:** Writing – review & editing, Resources, Funding acquisition. **Alexander Weigard:** Writing – review & editing. **Omid Kardan:** Writing – review & editing. **McCurry Katherine:** Writing – review & editing. **Chandra Sripada:** Writing – review & editing, Writing – original draft, Visualization, Resources, Methodology, Investigation, Funding acquisition, Conceptualization. **M. Fiona Molloy:** Writing – review & editing, Writing – original draft, Methodology, Investigation, Formal analysis, Conceptualization. **Aman Taxali:** Writing – original draft, Visualization, Resources, Methodology, Formal analysis, Data curation. **Mike Angstadt:** Writing – original draft, Visualization, Resources, Methodology, Formal analysis, Data curation. **Katherine Toda-Thorne:** Writing – review & editing.

## Declaration of Competing Interest

The authors declare that they have no known competing financial interests or personal relationships that could have appeared to influence the work reported in this paper.

## Figures and Tables

**Table:** 

Author	Dataset (N)	Research Design	Functional Connectivity Definition	Sleep Measure/ Manipulation	Functional Connectivity Results
Shao et al. (2013)	N = 14	Experimental	Seed-based (bilateral thalamus)	36 h sleep deprivation	Sleep deprivation resulted in decreased functional connectivity within the right parahippocampal gyrus and multiple temporal and parietal regions (right middle temporal gyrus, bilateral superior frontal gyrus, right medial frontal gyrus, and bilateral middle temporal gyri).
Liu et al. (2014)	N = 22	Experimental	Whole-brain	34 h sleep deprivation	Topological properties in the occipital network, sensorimotor network, and frontoparietal network were most vulnerable to sleep deprivation.
Yeo et al. (2015)	N = 68	Experimental	Whole-brain	One night of sleep deprivation	Highly-connected networks (including some default regions) became less integrated and highly segregated networks (including default and attention networks) became less segregated during sleep deprivation.
Ben Simon et al. (2017)	N = 18	Experimental	Whole-brain	24 h sleep deprivation	Sleep deprivation resulted in a significant reduction in network modularity, specifically in the limbic, default mode, salience, and executive networks.
Nilsonne et al. (2017)	N = 56 (Stockholm Sleepy Brain Study)	Experimental	Whole-brain, seed-based (default mode network, temporoparietal network, amygdala, thalamus)	Partial sleep deprivation (3 h of total sleep)	Sleep deprivation resulted in increased global signal variability, but there were no significant changes in resting state networks.
Xu et al. (2018)	N = 37	Experimental	Whole-brain	36 h sleep deprivation	Time-averaging functional connectivity across specific regions and temporal properties of the functional connectivity states were strongly influenced after sleep deprivation, particularly in default mode and dorsal attention networks.
Chen et al. (2018)	N = 51	Experimental	ICA-defined dorsal and ventral default mode networks	24 h sleep deprivation	Functional connectivity decreased within the dorsal default mode network and increased within the ventral default mode network and between the ventral and dorsal default mode networks following sleep deprivation.
Cai et al. (2021)	N = 42	Experimental	Seed-based (ALFF-defined regions)	24 h sleep deprivation	Sleep deprivation caused significant changes in resting brain activity, and sustained attentional impairments could be predicted by altered frontal connectivity with key neural regions involved in stimulus processing, such as the thalamus and visual cortex.
Zhang et al. (2021)	N = 26	Experimental	Seed-based (bilateral nucleus accumbens)	36 h sleep deprivation	Sleep deprivation reduced functional connectivity between the left nucleus accumbens and anterior cingulate cortex, and between the anterior cingulate cortex and the right inferior frontal gyrus.
Qi et al. (2021a)	N = 26	Experimental	Seed-based (bilateral insula)	36 h sleep deprivation	Sleep deprivation increased insular connectivity with the prefrontal cortex and anterior cingulate cortex, and reduced insular connectivity with temporal, parietal, and occipital regions.
Wang et al. (2021)	N = 30	Experimental	Whole-brain	36 h sleep deprivation	Sleep deprivation produced significant reductions in functional connectivity in the putamen and precentral gyrus, as well as the caudate and postcentral gyrus.
Qi et al. (2021b)	N = 26	Experimental	Seed based (bilateral hypothalamus)	36 h sleep deprivation	Sleep deprivation resulted in enhanced functional connectivity between the hypothalamus, thalamus, anterior cingulate, insula, amygdala, and reduced functional connectivity between the hypothalamus and middle frontal gyrus.
Brooks et al. (2022)	N = 5566 (Adolescent Brain Cognitive Development Study)	Observational	Whole-brain	Parent report of youth sleep duration	Decreased sleep duration was associated with lower topological efficiency, flexibility, and robustness of visual, sensorimotor, attention, frontoparietal control, default mode and/or limbic networks.
Ning et al. (2022)	N = 30	Experimental	Whole-brain	24 h sleep deprivation	Sleep depreciation decreased between-network functional connectivity between the sensorimotor network and the default mode, frontoparietal, and ventral attention networks, and increased between-network functional connectivity between the dorsal attention network and the ventral attention and sensorimotor networks.
Tian et al. (2022)	N = 30	Experimental	Whole-brain	34 h sleep deprivation	Connectivity between the subcortical network (thalamus) and the sensorimotor network (paracentral lobule) significantly predicted sleepiness.
Yang et al. (2022)	N = 8323 (Adolescent Brain Cognitive Development Study)	Observational	Seed-based (Gordon networks and subcortical regions), whole brain	Parent report	Sufficient and insufficient sleep group differences in both directions in cortical and subcortical areas across the brain, with the functional connectivity differences found in subcortical regions.
Chen et al. (2023)	N = 19	Experimental	Seed-based (peak coordinates of regional homogeneity-significant brain regions), targets: whole-brain voxels	36 h sleep deprivation	Sleep deprivation led to a notable increase in regional homogeneity in the right postcentral gyrus, which was positively linked to changes in risk perception.
Li et al. (2023)	N = 12	Experimental	Voxel-level degree centrality	52 h sleep deprivation and 14 h recovery sleep	There was higher connectivity strength in the sensorimotor cortex but a decrease in strength in the default mode and subcortical network, these were associated with attentional deficiencies and sleepiness.
Mummaneni et al. (2023)	N = 898 (Human Connectome Project), N = 1274 (Adolescent Brain Cognitive Development Study)	Observational	Whole-brain	Self-reported sleep duration (HCP), Fitbit, and parent report sleep duration (ABCD)	HCP and ABCD shared 38 functional connections predicting more sleep. Stronger occipito-parietal connections were found in individuals who sleep less. Default mode and dorsal attention networks were related to sleep deprivation and disturbance.
(Zhang et al., 2023)	N = 11,878 (Adolescent Brain Cognitive Development Study)	Observational	Whole-brain	Parent-report of youth sleep duration	Youth with elevated within-default mode network resting-state functional connectivity showed intensified associations between stressful environments and impulsivity via shorter sleep duration.
Peng et al. (2024a)	N = 36	Experimental	Seed-based (ROIs from prior analyses of other resting state features)	24 h sleep deprivation	Increases in resting-state fMRI features were found in the right superior temporal gyrus, bilateral postcentral gyrus, left middle temporal gyrus, and left precuneus.
Feng et al. (2024)	N = 26	Experimental	Seed-based (6 thalamic subregions)	24 h of sleep deprivation	Altered functional connectivity between the thalamus subregions and the bilateral frontal pole, bilateral middle temporal gyrus, bilateral supramarginal gyrus, and left precuneus.
Peng et al. (2024b)	N = 16 males	Experimental	Seed-based (bilateral anterior cingulate cortices) to whole-brain voxels targets	36 h sleep deprivation	Increased connectivity observed between anterior cingulate cortices and the left angular gyrus, as well as the right hippocampus. Decreased anterior cingulate cortex connectivity with the right insular cortex, right paracingulate gyrus, and bilateral putamen.
Tian et al. (2024)	N = 30 (Tian et al. (2022)), N = 57 (Stockholm Sleepy Brain Study)	Experimental	Whole-brain	One night of sleep deprivation	Diversity enhancement of connector hub at control network and salience network after sleep deprivation.
Liu et al. (2024)	N = 86 (Stockholm Sleepy Brain Study)	Experimental	Whole-brain, Seed-based (language regions)	Partial sleep deprivation (3 h sleep)	Sleep deprivation was associated with decreases and increases found between language regions and multiple cortical and subcortical areas, sex differences found.
Chen et al. (2023)	N = 19	Experimental	Seed-based (peak coordinates of regional homogeneity-significant regions), targets: whole-brain voxels	36 h sleep deprivation	Sleep deprivation led to a notable increase in regional homogeneity in the right postcentral gyrus, which was positively linked to changes in risk perception.
Weng et al. (2025)	N = 60	Experimental	Seed-based (pericalcarine cortex, inferior and middle occipital gyrus)	36 h sleep deprivation	Significant reductions in connectivity between the prefrontal cortex and visual processing regions and increased connectivity in the cuneus and lingual gyrus after sleep deprivation.
Yu et al. (2025)	N = 77 (Stockholm Sleepy Brain Study)	Experimental	Whole-brain	Partial sleep deprivation (3 h of sleep)	All participants showed decreased regional network strength in the default mode network. Older participants also had decreased strength in the dorsal attention network, and increased regional network strength in the visual network.
Turan et al. (2025)	N = 3799 (Adolescent Brain Cognitive Development Study)	Observational	Seed-based (12 Gordon functional networks)	Actigraphy (Fitbit)	Positive and negative correlations were found between and within networks across the brain including attention, sensorimotor, memory and executive control networks.
Zhang et al. (2025)	N = 36	Experimental	Whole-brain	24 h sleep deprivation	Disruption in networks involving the visual, sensorimotor, dorsal attention, and, to a lesser degree, the default mode network.
Qi and Hu (2025)	N = 77 (Stockholm Sleepy Brain Study)	Experimental	Seed-based (bilateral hypothalamus)	Partial sleep deprivation (3 h of sleep)	Young adults exhibited enhanced hypothalamic functional connectivity with the inferior frontal gyrus and precuneus, while older adults exhibited reduced functional connectivity between the hypothalamus and frontal, parietal, temporal, occipital regions, and cingulate gyrus.

## Data Availability

The authors do not have permission to share data.
