# Supplementary material for: Decreased sleep is linked longitudinally and directionally to alterations in the brain’s intrinsic functional architecture
Source: Dev Cogn Neurosci. 2025 Dec 31;78:101668. doi: 10.1016/j.dcn.2025.101668 (PMC12813488; doi:10.1016/j.dcn.2025.101668)
Supplement: Supplementary file 1 — Supplementary material [file mmc1.docx]

**Supplemental Methods and Results**

**1. Sample and Data**

The ABCD study is a multisite longitudinal study with 11,878 children between 9-10 years of age from 21 sites across the United States. The study conforms to the rules and procedures of each site’s Institutional Review Board, and all participants provide informed consent (parents) or assent (children). Data for this study are from ABCD Release 5.1.

**2. Data Acquisition, fMRI Preprocessing, and Connectome Generation**

Imaging protocols were harmonized across sites and scanners. High spatial (2.4mm isotropic) and temporal resolution (TR = 800ms) resting-state fMRI was acquired in four separate runs (5min per run, 20min total). The entire data pipeline described below was run through automated scripts on the University of Michigan’s high-performance cluster, and is described below.

Preprocessing was performed using fMRIPrep version 1.5.0[^1^](https://www.zotero.org/google-docs/?VyJbkB). T1-weighted (T1w) and T2-weighted images were run through recon-all using FreeSurfer v6.0.1. T1w images were also spatially normalized nonlinearly to MNI152NLin6Asym space using ANTs 2.2.0. Each functional run was corrected for fieldmap distortions, rigidly coregistered to the T1, motion corrected, and normalized to standard space. ICA-AROMA was run to generate aggressive noise regressors. Anatomical CompCor was run and the top 5 principal components of both CSF and white matter were retained. Functional data were transformed to CIFTI space using HCP’s Connectome Workbench. All preprocessed data were visually inspected at two separate stages to ensure only high-quality data was included: after co-registration of the functional data to the structural data and after registration of the functional data to MNI template space.

Connectomes were generated for each functional run using the Gordon 333 parcel atlas[^2^](https://www.zotero.org/google-docs/?HGJ5rf), augmented with parcels from high-resolution subcortical[^3^](https://www.zotero.org/google-docs/?JsYqNV) and cerebellar[^4^](https://www.zotero.org/google-docs/?cDSJUJ) atlases. Volumes exceeding a framewise displacement threshold of 0.5mm were marked to be censored. Covariates were regressed out of the time series in a single step, including: linear trend, 24 motion parameters (original translations/rotations + derivatives + quadratics), aCompCorr 5 CSF and 5 WM components and ICA-AROMA aggressive components, high-pass filtering at 0.008Hz, and censored volumes. Next, correlation matrices were calculated for each run. Each matrix was then Fisher r-to-z transformed, and then averaged across runs for each participant to yield their final connectome.

**3. Inclusion/Exclusion**

There are 11,878 participants in the ABCD Release 5.1 dataset. Screening was initially done using ABCD raw QC to limit to participants with 2 or more good runs of resting data as well as a good T1 and T2 image (QC score, protocol compliance score, and complete all = 1). After preprocessing each run was visually inspected for registration and warping quality, and only those participants who still had 2 or more good runs were retained. After connectome generation, runs were excluded if they had less than 4 minutes of uncensored data, and next participants were retained only if they had 2 or more good runs. Two ABCD samples and one Stockholm Sleepy Brain Study sample were employed in this study.

For the ABCD Discovery Sample, we focused on year-2 subjects who had parent-reported, child-reported, and objective sleep data for calculating a sleep factor. All participants who had the necessary measures for calculating the sleep factor at the year 2 timepoint and had values for nuisance covariates were included in the discovery sample for the multivariate neurosignature (N = 2,991).

For the ABCD Longitudinal Sample, we included all remaining participants who were not included in the Discovery Sample, had good data at both the baseline and year 2 visit (N = 1,574), and had parent reported-sleep and values for nuisance covariates. Demographic characteristics of the two ABCD samples are shown in **Table S1**.

The Stockholm Sleepy Brain study involved a within-subject sleep deprivation manipulation and was used to assess directional relationships between reduced sleep and brain connectivity. This sample contained 86 adults scanned on 2 days with one day being after a night of normal sleep and the other after a night of sleep deprivation (instructed to sleep for 3 hours). Subjects were included if they had ≥4 minutes after censoring frames with FD>0.5mm in both runs. This left N=76 participants for the Stockholm analysis. The included subjects were 51.3% female (N=39). They were 55.3% young (N=42, young is 20-30 years old, the remaining N=34 were 65-75 years old). Education level was collected as well: N=3 finished primary school, N=22 finished secondary school, N=22 university student, N=29 finished university.

|  |  | Longitudinal Sample | |
| --- | --- | --- | --- |
|  | Discovery Sample | Baseline | Year 2 |
| N | 2991 | 1574 | 1574 |
| Age (mean (s.d.)) | 11.95 (0.65) | 9.94 (0.63) | 12.02 (0.66) |
| Female (%) | 1502 (50.2) | 740 (47.0) | 740 (47.0) |
| Race (%) |  |  |  |
| White | 2162 (72.3) | 1066 (67.7) | 1066 (67.7) |
| Black | 267 (8.9) | 238 (15.1) | 238 (15.1) |
| Asian | 57 (1.9) | 28 (1.8) | 28 (1.8) |
| Multi-racial/Other | 505 (16.9) | 242 (15.4) | 242 (15.4) |
| No answer | – | – | – |
| Ethnicity (%) |  |  |  |
| Hispanic | 575 (19.2) | 277 (17.6) | 277 (17.6) |
| Highest Parental Education (%) |  |  |  |
| < HS Diploma | 45 (1.5) | 53 (3.4) | 47 (3.0) |
| HS Diploma/GED | 212 (7.1) | 147 (9.3) | 169 (10.7) |
| Some College | 701 (23.4) | 439 (27.9) | 409 (26.0) |
| Bachelor | 861 (28.8) | 402 (25.5) | 395 (25.1) |
| Post Graduate Degree | 1169 (39.1) | 532 (33.8) | 551 (35.0) |
| No answer | 3 (0.1) | 1 (0.1) | 3 (0.2) |
| Household Marital Status – Married (%) | 2194 (73.4) | 1067 (67.8) | 1061 (67.4) |
| Household Income (%) |  |  |  |
| <50K | 543 (18.2) | 470 (29.9) | 373 (23.7) |
| >=50k & <100K | 791 (26.4) | 474 (30.1) | 450 (28.6) |
| >=100k | 1508 (50.4) | 551 (35.0) | 642 (40.8) |
| No answer | 149 (5.0) | 79 (5.0) | 109 (6.9) |

***Table S1. Demographic Characteristics of Included Participants***

|  |  |
| --- | --- |
|  | Stockholm Sample |
| N | 76 |
| Age Group - Old (%) | 44.7% |
| Female (%) | 51.3% |
| Highest Education (%) |  |
| Completed Primary | 3.9% |
| Completed Secondary | 28.9% |
| Student at University | 38.2% |
| University Degree | 28.9% |

***Table S2. Demographic Characteristics of Included Stockholm Participants***

**4. Principal Components Regression-Based Multivariate Predictive Modeling**


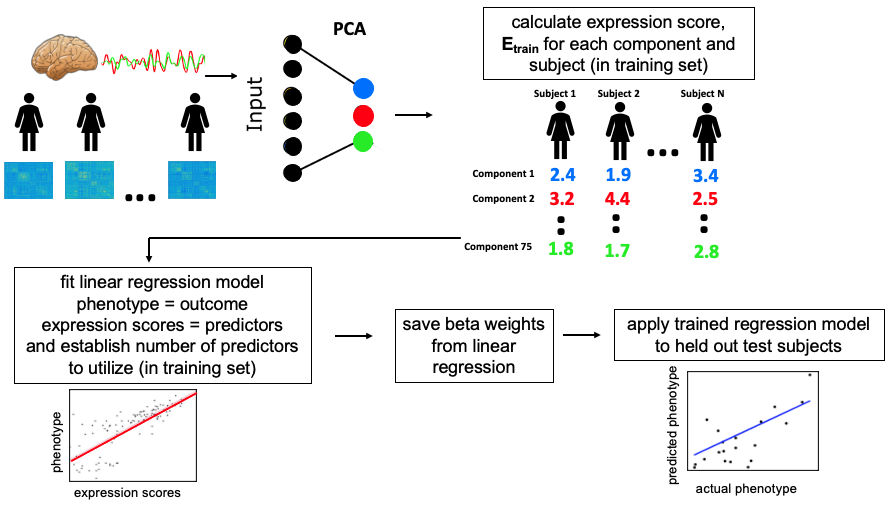


***Figure S1. Steps of Principal Component Regression Predictive Modeling.***

We implemented principal component regression (PCR)[^7^](https://www.zotero.org/google-docs/?lVJDsH) as a multivariate predictive modeling method for identifying brain-behavior relationships[^8^](https://www.zotero.org/google-docs/?uMiycZ) (see **Figure S1**). The method involves two key steps: 1) Use principal component analysis (PCA) to find a set of components that capture *inter-individual* differences in brain features; 2) Use multiple regression in a cross-validation framework to link expression scores for these components to phenotypes of interest. In previous work, we often used the more general name brain basis set (BBS) for this approach to capture commonalities with work by our group and others that use alternative methods for step 1 (e.g., independent component analysis[^9,10^](https://www.zotero.org/google-docs/?h7bOOf) or community detection[^11,12^](https://www.zotero.org/google-docs/?9fCUH4)). We chose the PCR approach for this study because our previous work showed it has high test-retest reliability[^13^](https://www.zotero.org/google-docs/?zzP0t6) and predictive accuracy[^14,15^](https://www.zotero.org/google-docs/?Pi8I9A) and generally performs as well as or better than alternative methods such as support vector regression and ridge regression[^13^](https://www.zotero.org/google-docs/?FZHaZN).

We performed PCA dimensionality reduction on an *n* subjects by *p* connectivity features matrix, yielding *n* principal components (i.e., directions in the feature space) that represent inter-individual differences in the imaging features (functional connectivity). Per-subject expression scores for a subset of *k* of these components then entered multiple regression modeling to identify linear associations with phenotypes of interest (here, sleep duration). Of note, we selected *k* using 5-fold cross-validation within the training data, as in our previous work[^13^](https://www.zotero.org/google-docs/?qgJGDq).

To assess accuracy and generalizability of PCR predictive models, we used leave-one-site-out cross-validation. In each fold of the cross-validation, data from one of the 21 sites served as the held-out test dataset and data from the other 20 sites served as the training dataset. Additionally, to ensure separation of train and test datasets, at each fold of the cross-validation, a new PCA was performed on the imaging features (functional connectivity) in the training dataset, and expression scores of these brain components were calculated for the test set. Note that by employing leave-one-site-out, members of twinships and sibships are never present in both training and test samples. We assessed the performance of PCR predictive models with cross-validated Pearson’s correlation and cross-validated partial eta squared.

In each fold of the leave-one-site out cross-validation (LOSO-CV), PCR predictive models were trained in the train partition with the following covariates (unless explicitly stated otherwise for specific analyses): sex, race, ethnicity, age, age squared, mean FD and mean FD squared. To maintain strict separation between training and test datasets, regression coefficients for the covariates learned from the training sample were applied to the test sample to calculate effect size measures (Pearson’s correlation_cross-validated_). This procedure is described in detail in our previous publications[^15,16^](https://www.zotero.org/google-docs/?iMsaWM).

We assessed the significance of all cross-validation-based correlations with non-parametric permutation tests. We randomly permuted the 2,991 subjects’ outcome variable values 10,000 times and reran the PCR predictive modeling stream at each iteration, yielding a null distribution of correlation values. The procedure of Freedman and Lane[^17^](https://www.zotero.org/google-docs/?59q5k4) was used to account for covariates. In addition, exchangeability blocks were used to account for twin, family, and site structure and were entered into Permutation Analysis of Linear Models (PALM)[^18^](https://www.zotero.org/google-docs/?YIaj3n) to produce permutation orderings.

**5. Latent Variable Modeling for Sleep Duration**

For the parent-reported Sleep Disturbance Scale (SDSC), we used the sleep duration item. This item asks “How many hours of sleep does your child get on most nights? 1 = 9-11 hours; 2 = 8-9 hours; 3 = 7-8 hours; 4 = 5-7 hours; 5 = Less than 5 hours. For each response, we assigned the midpoint of the associated range, and assigned 5 for the “Less than 5 hours response”. For the youth-rated Munich Chronotype Questionnaire, we determined sleep duration as the difference between (a) mctq_sow_calc (Sleep onset, workday) and mctq_sd_wake_up_time_calc (Sleep end, workday) and (b) mctq_sof_calc (Sleep onset, freeday) and mctq_fd_wake_up_time_calc (Sleep end, freeday), and calculated a weighted average. For the Fitbit data, we used the fit_ss_sleepperiod_minutes variable during both weekends and weekdays, and calculated a weighted average.

**6. Sensitivity Analysis with Race/Ethnicity as a Covariate**

The following section reports the results when race/ethnicity was included as a covariate. First, in the sample of 3,991 children from the ABCD year-2 study, the out-of-sample multivariate link between shorter sleep duration and brain-wide functional connectivity was r_cv_ = 0.2174 (averaging across sites). Second, in the longitudinal analysis of 1,574 ABCD children, changes in sleep duration were highly significantly associated with changes in expression of the reduced sleep duration neurosignature (standardized beta=0.10, *p*=0.00003).

Third, in the Stockholm sleepy brain study the expression of the ABCD reduced sleep neurosignature was significantly increased after sleep deprivation (mean=2.451, SE=0.339) compared to after a typical night of sleep (mean=1.097, SE=0.344), and the difference was highly statistically significant when controlling for sex, age group, and motion on each scan (T=3.52, p=0.0008). Finally, the spatial correlation across connections between the ABCD and Stockholm neurosignatures was 0.37, *p*_PERM_=0.0076. These results are highly similar to the results reported in the main paper, and none of the conclusions change when including race/ethnicity as a covariate.

**7. Sensitivity Analysis with Average Sleep**

The following section reports the results when the outcome variable was a simple average of z-scored sleep metrics, rather than the latent variable. First, in the sample of 3,991 children from the ABCD year-2 study, the out-of-sample multivariate link between shorter sleep duration and brain-wide functional connectivity was r_cv_ = 0.3190 (averaging across sites). Second, in the longitudinal analysis of 1,574 ABCD children, changes in sleep duration were highly significantly associated with changes in expression of the reduced sleep duration neurosignature (standardized beta=0.10, *p*=0.00005).

Third, in the Stockholm sleepy brain study the expression of the ABCD reduced sleep neurosignature was significantly increased after sleep deprivation (mean=2.716, SE=0.343) compared to after a typical night of sleep (mean=1.354, SE=0.344), and the difference was highly statistically significant when controlling for sex, age group, and motion on each scan (T=3.56, p=0.0007). Finally, the spatial correlation across connections between the ABCD and Stockholm neurosignatures was 0.37, *p*_PERM_=0.0081. These results are highly similar to the results reported in the main paper, and none of the conclusions change when using the sleep factor as a covariate.

**References**

[1. Esteban, O. *et al.* fMRIPrep: a robust preprocessing pipeline for functional MRI. *Nat. Methods* **16**, 111–116 (2019).](https://www.zotero.org/google-docs/?0ftjQB)

[2. Gordon, E. M. *et al.* Generation and Evaluation of a Cortical Area Parcellation from Resting-State Correlations. *Cereb. Cortex* **26**, 288–303 (2016).](https://www.zotero.org/google-docs/?0ftjQB)

[3. Tian, Y., Margulies, D. S., Breakspear, M. & Zalesky, A. Topographic organization of the human subcortex unveiled with functional connectivity gradients. *Nat. Neurosci.* **23**, 1421–1432 (2020).](https://www.zotero.org/google-docs/?0ftjQB)

[4. Diedrichsen, J. *et al.* Imaging the deep cerebellar nuclei: A probabilistic atlas and normalization procedure. *NeuroImage* **54**, 1786–1794 (2011).](https://www.zotero.org/google-docs/?0ftjQB)

[5. Power, J. D. *et al.* Methods to detect, characterize, and remove motion artifact in resting state fMRI. *NeuroImage* **84**, 320–341 (2014).](https://www.zotero.org/google-docs/?0ftjQB)

[6. Power, J. D., Schlaggar, B. L. & Petersen, S. E. Recent progress and outstanding issues in motion correction in resting state fMRI. *NeuroImage* **105**, 536–551 (2015).](https://www.zotero.org/google-docs/?0ftjQB)

[7. Jolliffe, I. T. A Note on the Use of Principal Components in Regression. *J. R. Stat. Soc. Ser. C Appl. Stat.* **31**, 300–303 (1982).](https://www.zotero.org/google-docs/?0ftjQB)

[8. Sripada, C. *et al.* Basic Units of Inter-Individual Variation in Resting State Connectomes. *Sci. Rep.* **9**, 1900 (2019).](https://www.zotero.org/google-docs/?0ftjQB)

[9. Kessler, D., Angstadt, M. & Sripada, C. Growth Charting of Brain Connectivity Networks and the Identification of Attention Impairment in Youth. *JAMA Psychiatry* **73**, 481–489 (2016).](https://www.zotero.org/google-docs/?0ftjQB)

[10. Kessler, D., Angstadt, M., Welsh, R. C. & Sripada, C. Modality-spanning deficits in attention-deficit/hyperactivity disorder in functional networks, gray matter, and white matter. *J. Neurosci. Off. J. Soc. Neurosci.* **34**, 16555–16566 (2014).](https://www.zotero.org/google-docs/?0ftjQB)

[11. Arroyo, J. *et al.* Inference for Multiple Heterogeneous Networks with a Common Invariant Subspace. *J. Mach. Learn. Res.* **22**, 1–49 (2021).](https://www.zotero.org/google-docs/?0ftjQB)

[12. Levin, K., Lodhia, A. & Levina, E. Recovering shared structure from multiple networks with unknown edge distributions. *arXiv.org* (2021).](https://www.zotero.org/google-docs/?0ftjQB)

[13. Taxali, A., Angstadt, M., Rutherford, S. & Sripada, C. Boost in Test–Retest Reliability in Resting State fMRI with Predictive Modeling. *Cereb. Cortex* **31**, 2822–2833 (2021).](https://www.zotero.org/google-docs/?0ftjQB)

[14. Sripada, C. *et al.* Prediction of neurocognition in youth from resting state fMRI. *Mol. Psychiatry* **25**, 3413–3421 (2020).](https://www.zotero.org/google-docs/?0ftjQB)

[15. Sripada, C., Angstadt, M., Rutherford, S., Taxali, A. & Shedden, K. Toward a ‘treadmill test’ for cognition: Improved prediction of general cognitive ability from the task activated brain. *Hum. Brain Mapp.* **41**, 3186–3197 (2020).](https://www.zotero.org/google-docs/?0ftjQB)

[16. Sripada, C. *et al.* Brain-wide functional connectivity patterns support general cognitive ability and mediate effects of socioeconomic status in youth. *Transl. Psychiatry* **11**, 1–8 (2021).](https://www.zotero.org/google-docs/?0ftjQB)

[17. Freedman, D. & Lane, D. A Nonstochastic Interpretation of Reported Significance Levels. *J. Bus. Econ. Stat.* **1**, 292–298 (1983).](https://www.zotero.org/google-docs/?0ftjQB)

[18. Winkler, A. M., Ridgway, G. R., Webster, M. A., Smith, S. M. & Nichols, T. E. Permutation inference for the general linear model. *NeuroImage* **92**, 381–397 (2014).](https://www.zotero.org/google-docs/?0ftjQB)

[19. Rakesh, D., Zalesky, A. & Whittle, S. Similar but distinct – Effects of different socioeconomic indicators on resting state functional connectivity: Findings from the Adolescent Brain Cognitive Development (ABCD) Study®. *Dev. Cogn. Neurosci.* **51**, 101005 (2021).](https://www.zotero.org/google-docs/?0ftjQB)

[20. Brislin, S. J. *et al.* Differentiated nomological networks of internalizing, externalizing, and the general factor of psychopathology (‘p factor’) in emerging adolescence in the ABCD study. *Psychol. Med.* **52**, 3051–3061 (2022).](https://www.zotero.org/google-docs/?0ftjQB)
